# Two new replacement names for the planthopper genera in Dictyopharidae (Hemiptera, Fulgoromorpha)

**DOI:** 10.3897/zookeys.309.5183

**Published:** 2013-06-14

**Authors:** Jichun Xing, Xiangsheng Chen

**Affiliations:** 1 Institute of Entomology, Guizhou University; The Provincial Key Laboratory for Agricultural Pest Management of Mountainous Region, Guiyang, Guizhou, P. R. China, 550025

**Keywords:** Homoptera, Dictyopharidae, planthopper, homonym, replacement name

## Abstract

New replacement names are proposed for two genera of the family Dictyopharidae (Hemiptera: Fulgoromorpha). The following changes are proposed: *Neonotostrophia* **nom. n.** for *Notostrophia* Emeljanov (not Waterhouse); *Emeljanovina* **nom. n.** for *Glochina* Emeljanov (not Meigen); *Neonotostrophia nigrosuturalis* (Melichar, 1912) **comb. n.** from *Notostrophia nigrosuturalis* (Melichar, 1912) = *Dictyophara nigrosuturalis* Melichar, 1912 and *Emeljanovina dixoni* (Distant, 1906) **comb. n.** from *Glochina dixoni* (Distant, 1906) = *Dictyophara dixoni* Distant, 1906.

## Introduction

The purpose of the present paper is to bring the taxonomy of planthoppers into accordance with the International Code of Zoological Nomenclature (1999). Two homonyms were discovered for genera names in Dictyopharidae (Hemiptera: Fulgoromorpha). In an effort to reduce homonyms in Fulgoromorpha, we propose two replacement names for these genera.

## Nomenclatural changes and notes

### Genus Neonotostrophia nom. n.

Notostrophia Emeljanov, 2011: 305 (Insecta: Hemiptera: Fulgoroidea: Dictyopharidae). Preoccupied by Waterhouse 1973: 35 (Brachiopoda: Strophomenata: Orthotetida: Schuchertellidae).

#### Type species.

*Dictyophara nigrosuturalis* Melichar, 1912.

#### Remarks on nomenclatural change.

Emeljanov (2011) established the planthopper genus *Notostrophia* with the type species *Dictyophara nigrosuturalis* Melichar, 1912. So far, this genus includes only the type species. This genus is used as a valid generic name in Dictyopharidae. Unfortunately, the name *Notostrophia* Emeljanov (2011) was preoccupied by *Notostrophia* Waterhouse (1973), a genus of Schuchertellidae (Brachiopoda: Strophomenata: Orthotetida) based on type species *Notostrophia homeri* Waterhouse, 1973. Thus, the planthopper genus *Notostrophia* Emeljanov, 2011 is a junior homonym of the genus *Notostrophia* Waterhouse, 1973. According to Article 60 of the International Code of Zoological Nomenclature, we propose a new replacement name *Neonotostrophia* nom. n. for *Notostrophia* Emeljanov, 2011.

#### Etymology.

From the preexisting name *Notostrophia*, the prefix “Neo-” from the Greek “*neos*” meaning new; gender feminine.

#### Summary of nomenclatural changes.

*Neonotostrophia* new replacement name = *Notostrophia* Emeljanov, 2011 (nec Waterhouse 1973)

*Neonotostrophia nigrosuturalis* (Melichar, 1912) comb. n. = *Notostrophia nigrosuturalis* (Melichar, 1912) = *Dictyophara nigrosuturalis* Melichar, 1912

### Genus Emeljanovina nom. n.

Glochina Emeljanov, 2011: 320 (Hemiptera: Fulgoroidea: Dictyopharidae). Preoccupied by Meigen 1830: 280 (Diptera: Tipuloidea: Limoniidae).

#### Type species.

*Dictyophara dixoni* Distant, 1906.

#### Remarks on nomenclatural change.

Emeljanov (2011) established the planthopper genus *Glochina* with the type species *Dictyophara dixoni* Distant, 1906. So far, this genus includes only type species. It is used as a valid generic name. Unfortunately, the name *Glochina* Emeljanov (2011) was preoccupied by *Glochina* Meigen (1830), a subgenus *Dicranomyia (Glochina)* Meigen, 1830 in Limoniidae (Diptera: Tipuloidea) based on type species *Glochina sericata* Meigen, 1830. Thus, the genus *Glochina* Emeljanov, 2011 is a junior homonym of the subgenus *Dicranomyia (Glochina)* Meigen, 1830. According to Article 60 of the International Code of Zoological Nomenclature, we propose a new replacement name *Emeljanovina* nom. n. for *Glochina* Emeljanov, 2011.

**Etymology.** The genus from A. F. Emeljanov who is the author of the preexisting *Glochina*; gender feminine.

#### Summary of nomenclatural changes.

*Emeljanovina* new replacement name = *Glochina* Emeljanov, 2011 (nec Meigen 1830) *Emeljanovina dixoni* (Distant, 1906) comb. n. = *Glochina dixoni* (Distant, 1906) = *Dictyophara dixoni* Distant, 1906.

## Supplementary Material

XML Treatment for Neonotostrophia

XML Treatment for Emeljanovina
